# Creating efficiencies in the extraction of data from randomized trials: a prospective evaluation of a machine learning and text mining tool

**DOI:** 10.1186/s12874-021-01354-2

**Published:** 2021-08-16

**Authors:** Allison Gates, Michelle Gates, Shannon Sim, Sarah A. Elliott, Jennifer Pillay, Lisa Hartling

**Affiliations:** Department of Pediatrics and the Alberta Research Centre for Health Evidence, University of Alberta, Edmonton Clinic Health Academy, 11405 87 Avenue NW, Edmonton, Alberta T6G 1C9 Canada

**Keywords:** Data collection, Machine learning, Text mining, Efficiency, Systematic reviews, Clinical trials

## Abstract

**Background:**

Machine learning tools that semi-automate data extraction may create efficiencies in systematic review production. We evaluated a machine learning and text mining tool’s ability to (a) automatically extract data elements from randomized trials, and (b) save time compared with manual extraction and verification.

**Methods:**

For 75 randomized trials, we manually extracted and verified data for 21 data elements. We uploaded the randomized trials to an online machine learning and text mining tool, and quantified performance by evaluating its ability to identify the reporting of data elements (reported or not reported), and the relevance of the extracted sentences, fragments, and overall solutions. For each randomized trial, we measured the time to complete manual extraction and verification, and to review and amend the data extracted by the tool. We calculated the median (interquartile range [IQR]) time for manual and semi-automated data extraction, and overall time savings.

**Results:**

The tool identified the reporting (reported or not reported) of data elements with median (IQR) 91% (75% to 99%) accuracy. Among the top five sentences for each data element at least one sentence was relevant in a median (IQR) 88% (83% to 99%) of cases. Among a median (IQR) 90% (86% to 97%) of relevant sentences, pertinent fragments had been highlighted by the tool; exact matches were unreliable (median (IQR) 52% [33% to 73%]). A median 48% of solutions were fully correct, but performance varied greatly across data elements (IQR 21% to 71%). Using ExaCT to assist the first reviewer resulted in a modest time savings compared with manual extraction by a single reviewer (17.9 vs. 21.6 h total extraction time across 75 randomized trials).

**Conclusions:**

Using ExaCT to assist with data extraction resulted in modest gains in efficiency compared with manual extraction. The tool was reliable for identifying the reporting of most data elements. The tool’s ability to identify at least one relevant sentence and highlight pertinent fragments was generally good, but changes to sentence selection and/or highlighting were often required.

**Protocol:**

https://doi.org/10.7939/DVN/RQPJKS

**Supplementary Information:**

The online version contains supplementary material available at 10.1186/s12874-021-01354-2.

## Background

Timely systematic reviews provide an indispensable resource for decision makers, many of whom lack the time and expertise to independently identify and evaluate new evidence. To be useful, systematic reviews must be conducted with a high degree of methodological rigor, and are therefore time and resource intensive. A typical systematic review will take a highly skilled team of clinician-experts, methodologists, and statisticians many months or even years to complete [1]. Especially in rapidly evolving fields, it is no longer feasible for traditional systematic review production to keep pace with the publication of new trial data, [2] seriously undermining the currency, validity, and utility of even the most recently published reviews.

As the number of newly registered randomized trials continues to grow, [3] the need to create efficiencies in the production of systematic reviews is increasingly pressing. Living systematic reviews, which are continually updated as new evidence becomes available, [4] represent a relatively new form of evidence synthesis aimed at addressing the heavy workload and fleeting currency associated with most traditional systematic reviews. Because living systematic reviews are updated in real time, the total workload for keeping them up to date is broken down into more manageable tasks [4]. Since living systematic reviews are held to the same methodological standards as traditional systematic reviews, the efficiency of their production will be critical to their feasibility and sustainability [4]

To date, nearly 200 software tools aimed at facilitating systematic review processes have been developed, with machine learning and text mining being the driver behind the proposed efficiencies of many tools [5]. Most research investigating the use of machine learning tools in systematic reviews has focused on creating efficiencies during the study selection step [6, 7]. The body of research investigating technologies designed to assist with data extraction, one of the most time- and resource-intensive steps of completing a systematic review, [8, 9] is comparatively immature [7, 10]. Machine learning tools that automatically identify relevant text may expedite data extraction in a number of ways: as a first check for manual data extraction performed in duplicate; to validate data extraction by a single reviewer; as the primary source for data extraction that would be validated by a human; and eventually to completely automate data extraction [7]

Among the tools that have been developed to semi-automate data extraction, few [11–13] prototypes have been made accessible for review teams to evaluate in practice [10]. Of the tools that are available, relatively few support semi-automated data extraction from full texts, [7, 10] and published evaluations of those that do are sparse [7]. Independent evaluations are needed to validate the relevance of automatically extracted data and potential for time and resource savings associated with using machine learning tools to assist with data extraction in systematic reviews.

### Objectives

We aimed to: (1) prospectively evaluate an online machine learning and text mining tool’s ability to automatically extract relevant data from randomized trials and (2) estimate the time savings associated with potential approaches to semi-automated data extraction compared with manual extraction and verification by two reviewers.

## Methods

### Machine learning and text mining tool

ExaCT (prototype available at https://exact.cluster.gctools.nrc.ca/ExactDemo/intro.php) is an online machine learning and text mining tool integrated within an automatic information extraction engine [13]. Developed jointly by the National Research Council of Canada and the University of California, San Francisco, the tool assists reviewers by automatically extracting study characteristics (hereafter referred to as “data elements”) from publications of randomized trials [13]. ExaCT was the first tool (and remains one of few tools) to automatically extract data from full text publications; various other tools extract data from abstracts only [7, 13]. Details of the design and development of ExaCT, and an early evaluation of its performance were reported in a 2010 publication by the tool’s developers [13]. Training of the tool occurred using a set of 132 full text articles extracted from 22 clinical journals (not restricted by clinical domain); the articles were selected due to their reasonably good reporting quality [13]. During training, a field expert manually annotated 78 of the articles (from 5 general medicine journals) to identify the target data elements [13]. Next, 54 articles from a larger pool of journals were added to the training set; training then occurred in a semi-supervised manner, whereby ExaCT automatically extracted the data elements, which were then revised by the field expert [13]. For the evaluation described herein, we used the publically available demo version of ExaCT, which does not require users to undertake additional training of the machine learning algorithm.

After creating an account, full texts can be uploaded to ExaCT’s user interface in HTML format. Nearly instantaneously, the tool extracts 21 unique data elements, as identified in sentences from each full text document. For each data element, the tool presents “solutions” consisting of five potentially relevant sentences presented in descending order of confidence. The top scoring sentence is termed the “system suggestion.” Text fragments (a word or group of words) that the system identifies as containing target information are highlighted within the retrieved sentences when the confidence score of those sentences exceeds a certain threshold. For each data element, the tool provides any of four responses: not found (i.e., data not reported and no relevant sentences); exactly one answer provided by one instance of text; one answer repeated in several instances of text; or several distinct answers. The tool allows users to view, confirm, refute, and modify the extracted sentences and text fragments.

Using a sample of 50 randomized trials published across 25 scientific journals, ExaCT’s developers reported 80% precision (i.e., the proportion of returned instances that are truly relevant) and recall (i.e., the proportion of relevant instances returned by the system) for extracted sentences [13]. Of the top five candidate sentences, the human reviewers considered at least one to be relevant 93% of the time [13]. With respect to the highlighted text fragments, on average the tool performed with 93% precision and 91% recall. It required the reviewer a mean 7 min and 21 s per trial publication to review ExaCT’s extracted data and make any necessary amendments. The authors did not measure time savings compared with extraction by human reviewers, acknowledging that a large-scale usability study is required to verify actual gains in efficiency [13]. Time savings attributed to the tool would result mainly from the reviewers being automatically directed to potentially relevant segments of text, expediting the identification and extraction of relevant information.

### Sample of Randomized Trials

We leveraged a random sample of randomized trials originally identified for an ongoing surveillance study that is underway at our center [14, 15]. On February 19, 2020 our research librarian undertook a search in the Cochrane Central Register of Controlled Trials (Wiley) for all child-relevant randomized trials of health interventions published in 2017 (Additional File 1). The search retrieved 17,703 potentially relevant citations, which we randomly ordered using the random numbers generator in Microsoft Excel. From the randomly ordered list, two independent reviewers (either of AG, MG, and SS) screened the titles and abstracts to identify the first 75 randomized trials that reported on outcomes for participants aged 21 years or younger (unrestricted by condition, intervention, comparator, or outcome type). Any record marked as “include” or “unsure” by either reviewer was eligible for scrutiny by full text. Two reviewers (either of AG, MG, and SS) independently screened the full texts and agreed upon the included randomized trials.

We selected our sample size for feasibility with respect to time, resources, and available personnel. The sample used for this study should have zero overlap with the developers’ test set, which included only randomized trials published in 2009 [13]. There should also be no overlap with their training set, which included only randomized trials published before 2010 [13]. One of two reviewers from a collaborating center extracted the study characteristics from each randomized trial.

### Data Collection

Three reviewers completed the data extraction following a three stage process, summarized in Additional File 2. All reviewers (AG, MG, and SS) hold postgraduate degrees in the health sciences and have substantial experience with data extraction and the conduct of systematic reviews. None of the reviewers were involved in the development or primary evaluation of the ExaCT tool. All reviewers were naïve to the tool prior to undertaking this study.

First, using the random numbers generator in Microsoft Excel, each reviewer was randomized to manually extract data from one-third (*n* = 25) of the sample of randomized trials and to verify the extracted data for a different one-third (*n* = 25) of randomized trials. Next, for their original sample of randomized trials, each reviewer collected data about the relevance of ExaCT’s automated extractions, as compared with their own verified extractions. The judgments were verified by a second reviewer. Finally, for the remaining 25 randomized trials to which they were naïve (i.e., had not yet reviewed for the purpose of data extraction or verification), each reviewer prospectively simulated semi-automated data extraction in ExaCT to measure time savings. This three stage process allowed us to control for gains in efficiency that would result from being familiar with the randomized trials.

Prior to beginning formal extraction, all reviewers pilot tested the data extraction forms on three randomized trials and convened to ensure a mutual understanding of the form, data elements, and timing procedure.

### A. Manual extraction and verification

For each randomized trial, the reviewers extracted ExaCT’s standard 21 data elements to a Microsoft Excel spreadsheet: eligibility criteria, sample size (enrolled), start date of enrollment, end date of enrollment, name of experimental treatment(s), name of control treatment(s), dose (or number of sessions), frequency of treatment, route of treatment (or delivery method), duration of treatment, primary outcome name, primary outcome time point, secondary outcome name, secondary outcome time point, funding organization name, funding number, early stopping, registration number, author name, date of publication, and digital object identifier (DOI). A second reviewer verified the extraction. The reviewers used a digital chronograph to measure the amount of time required to extract the data and verify the extractions, to the nearest 5 s. The timing began when the reviewer started reading the full text to extract or verify the data elements, and ended when the final data element was extracted or verified.

### B. Relevance of the automated extraction

For the same sample of randomized trials each reviewer reviewed the automatically extracted sentences and text fragments for each data element and judged the relevance of the sentences, highlighted text fragments, and overall solutions. For the purpose of this study, the data manually extracted by one reviewer and verified by another served as the reference standard. Although human reviewer judgment is imperfect, [16] dual independent extraction is recommended by leaders in evidence synthesis [17] and provided a reasonable standard for comparison. A second reviewer verified the judgments, and all disagreements were resolved through discussion.

At the sentence level, for each data element the reviewers judged whether the top-ranked sentence was relevant (yes or no) and whether at least one sentence was relevant (even if it was not the top-ranked sentence; yes or no). At the fragment level, for each sentence that the reviewer considered relevant, they judged whether the highlighted text fragments were fully or at least partially relevant (yes or no) [13]. Fully relevant fragments were those that encompassed all relevant information for the data element, without including additional irrelevant information or missing critical information. Partially relevant fragments were those that encompassed part of the relevant information, but either also included erroneous information or fell short of including all essential details. Additional File 3 shows examples of relevant and irrelevant sentences, and relevant, irrelevant, and partially relevant fragments.

To evaluate the relevance of the overall solutions, for each data element the reviewers recorded the number of fully relevant, partially relevant, and fully irrelevant solutions [13]. The relevance of the overall solutions accounts for the tool’s judgment of the reporting of the data element (reported or not reported), as well as relevance of the extracted sentences and fragments. Fully relevant solutions were those where the tool (a) correctly identified that the data element was reported, and the top sentence and its highlighted fragment(s) were relevant, or (b) correctly identified that the data element was not reported (i.e., returned a “not found” solution). Partially relevant solutions were those where the correct solution was present among the five sentences, but not (only) in the top sentence and/or the fragment selection in the sentence(s) was not entirely relevant. Fully irrelevant solutions were those where (a) none of the five suggested sentences contained relevant information pertaining to the data element, or (b) the data element was incorrectly identified as reported or not reported. Additional File 3 shows examples of fully relevant, partially relevant, and fully irrelevant solutions.

### C. Time savings

To measure the time saved by using ExaCT to assist with data extraction, the three reviewers examined the automatically extracted data elements and undertook necessary amendments, simulating a practical use of the tool. As with manual extraction, the reviewers used a digital chronograph to measure the time required to review and amend the automatically extracted data elements to the nearest 5 s. Timing began once the data extraction form was opened on the user interface and ended once all data elements were verified, revised, and downloaded.

### Data analysis

We synthesized the trial characteristics, the relevance of the extracted sentences, fragments, and overall solutions, and the timing data using descriptive statistics (counts, frequencies, median and interquartile range [IQR]). We presented the findings for the relevance of the automated extractions at the level of the randomized trials (i.e., medians and IQRs for all 21 data elements in each trial, across the 75 trials) and at the level of the individual data elements (i.e., medians and IQRs for each data element, across the 75 trials). We compared the time to complete the manual data extraction and verification with the time to complete the semi-automated extraction and interpreted differences with respect to practical significance. We calculated the time savings for two potential uses of ExaCT: (a) to assist the first reviewer in a pair, and (b) to replace the first reviewer in a pair. We calculated time savings as follows:

- *If ExaCT were used to assist the first reviewer in a pair:*
  Time savings = (time the first reviewer spent manually extracting data from the randomized trials) – (time one reviewer spent reviewing and amending ExaCT’s extractions).
  Note that the time savings here applies only to the work of the first reviewer in a pair. For the purpose of this study, we have assumed that the work of the second reviewer (verification) would remain constant.
- *If ExaCT were used to replace the first reviewer in a pair:*

Time savings = (time the two reviewers spent manually extracting and verifying data from the randomized trials) – (time one reviewer spent reviewing and amending ExaCT’s extractions).

## Results

### Sample of randomized trials

The included randomized trials are listed in Additional File 4 and summary characteristics of the sample are in Table 1. Nearly all (*n* = 70/75, 93.3%) randomized trials were efficacy or superiority trials. Most randomized trials used either a parallel (*n* = 54/75, 72.0%) or cluster (*n* = 14/75, 18.7%) design. The most common interventions included drugs (*n* = 18/75, 24.0%), rehabilitation or psychosocial programs (*n* = 12/75, 16.0%), communication, organizational, or educational programs (*n* = 12/75, 16.0%), and medical devices (*n* = 11/75, 14.7%). Nearly half (*n* = 36/75, 48.0%) used an active control, 20.0% (*n* = 15/75) used a placebo, 20.0% (*n* = 15/75) used a no intervention control, and 12.0% used a wait-list control. The primary outcome was most commonly a measure of physiological (*n* = 22/75, 29.3%), behavioral (*n* = 16/75, 21.3%), or psychological (*n* = 13/75, 17.3%) health, or a biomarker (e.g., serum ferritin, glycosylated hemoglobin) (*n* = 12/75, 16.0%).

**Table 1:** Summary characteristics of the sample of trials (*n* = 75)

Characteristic	Category	n (%)
**Study type**	Efficacy/superiority	70 (93.3)
	Equivalence	4 (5.3)
	Noninferiority	1 (1.3)
**Trial design**	Parallel	54 (72.0)
	Cluster	14 (18.7)
	Crossover	3 (4.0)
	Split body	2 (2.7)
	Factorial	0 (0)
	Other	2 (2.7)
**Intervention class**	Drug	18 (24.0)
	Communication, organizational, or educational	12 (16.0)
	Rehabilitation or psychosocial	12 (16.0)
	Device	11 (14.7)
	Alternative therapeutic	7 (9.3)
	Prevention or screening	6 (8.0)
	Vaccine	3 (4.0)
	Surgery or radiotherapy	2 (2.7)
	Other	4 (5.3)
**Control type**	Active intervention	36 (48.0)
	No intervention	15 (20.0)
	Placebo	15 (20.0)
	Wait-list control	9 (12.0)
**Primary outcome category**	Physiological	22 (29.3)
	Behavioral	16 (21.3)
	Psychological	13 (17.3)
	Biomarker	12 (16.0)
	Techniques or training	5 (6.7)
	Quality of life	2 (2.7)
	Pain	1 (1.3)
	Other	4 (5.3)

### A. Manual extraction and verification

On the basis of the human reviewers’ manual extractions, the reporting of the 21 data elements varied across the randomized trials (Table 2). Eligibility criteria, sample size, the experimental and control arms, and primary outcome(s) were reported in all 75 randomized trials. The primary outcome time point was reported in all but one randomized trial (*n* = 74/75, 98.7%). The funding source (*n* = 63/75, 84.0%), registration number (*n* = 52/75, 69.3%), enrollment start and end dates (*n* = 45/75, 60.0%), secondary outcome(s) (*n* = 55/75, 73.3%), and secondary outcome time point (*n* = 54/75, 72.0%) were reported in the majority of randomized trials. The funding number (*n* = 29/75, 38.7%) and early stopping (*n* = 4, 5.3%) were infrequently reported. Because of the nature of the interventions in this sample of randomized trials, the route of administration (*n* = 29/75, 38.7%) and dose (*n* = 37/75, 49.3%) were frequently irrelevant and not reported. The frequency (*n* = 43/75, 57.3%) and duration (*n* = 55/75, 73.3%) of the intervention were more frequently reported.

**Table 2:** Relevance of the automatically extracted sentences

Report section	Data element	Reported in the trial, n (%)^a^	Found by Exact, n (%)^b^	Relevance, top sentence, n (%)^c^	Relevance, any sentence, n (%)^c^	Relevant sentences, n (%) of Total
**Publication information**	First author name	75 (100.0)	74 (98.7)	63 (85.1)	n/a	n/a
	Date of publication	75 (100.0)	74 (98.7)	64 (86.5)	n/a	n/a
	Digital object identifier	75 (100.0)	72 (96.0)	62 (82.7)	n/a	n/a
**Meta information**	Funding source	63 (84.0)	58 (92.1)	45 (77.6)	50 (86.2)	79/116 (68.1)
	Funding number	*29 (38.7)*	22 (75.9)	20 (90.9)	22 (100.0)	35/110 (31.8)
	Registration number	52 (69.3)	40 (76.9)	40 (100.0)	40 (100.0)	63/200 (31.5)
**Enrollment**	Eligibility criteria	75 (100.0)	75 (100.0)	*38 (50.7)*	*47 (62.7)*	*110/375 (29.3)*
	Sample size	75 (100.0)	68 (90.7)	*32 (47.1)*	*43 (63.2)*	125/340 (36.8)
	Enrollment start date	*45 (60.0)*	44 (97.8)	35 (79.5)	44 (100.0)	*55/220 (25.0)*
	Enrollment end date	*45 (60.0)*	45 (100.0)	35 (77.8)	44 (97.8)	*56/225 (24.9)*
	Early stopping	*4 (5.3)*	*2 (50.0)*	2 (100.0)	2 (100.0)	7/10 (70.0)
**Intervention**	Experimental arm(s)	75 (100.0)	74 (98.7)	*43 (58.1)*	65 (87.8)	123/370 (33.2)
	Control arm(s)	75 (100.0)	75 (100.0)	49 (65.3)	65 (86.7)	121/375 (32.3)
	Route of administration	*29 (38.7)*	*14 (48.3)*	12 (85.7)	14 (100.0)	32/70 (45.7)
	Dose	*37 (49.3)*	32 (86.5)	*19 (59.4)*	28 (87.5)	50/160 (31.3)
	Frequency of administration	*43 (57.3)*	*28 (65.1)*	23 (82.1)	27 (96.4)	45/140 (32.1)
	Duration of treatment	55 (73.3)	*41 (74.5)*	*25 (61.0)*	*30 (73.2)*	*57/205 (27.8)*
**Outcome**	Primary outcome(s)	75 (100.0)	75 (100.0)	53 (70.7)	62 (82.7)	*95/375 (25.3)*
	Primary outcome time point	74 (98.7)	*50 (67.6)*	*27 (54.0)*	*39 (78.0)*	76/250 (30.4)
	Secondary outcome(s)	55 (73.3)	44 (80.0)	33 (75.0)	40 (90.9)	75/220 (34.1)
	Secondary outcome time point	54 (72.0)	*23 (42.6)*	15 (65.2)	*19 (82.6)*	43/115 (37.4)
**Summary measure**	Median (IQR), n	**55 (45 to 75)**	**44 (23 to 68)**	**35 (23 to 45)**	**40 (23 to 44)**	**57 (37 to 91)**
	Median (IQR), %	**73.3 (60.0 to 100.0)**	**90.7 (74.5 to 98.7)**	**77.6 (61.0 to 85.1)**	**87.7 (82.6, 99.5)**	**32.0 (29.3 to 36.1)**

*IQR*  Interquartile range, *n/a* Not applicable (ExaCT presents only one solution for these elements). Values in *italics* typeface fall at or below the limit of the lowest quartile

^a^As identified during manual data extraction and verification

^b^Pertains to the studies where the data element was identified as reported in the study by the human reviewers (denominator, column 3)

^c^Pertains to the studies where the data element was correctly identified as reported in the study by ExaCT (denominator, column 4)

### B. Relevance of the automated extraction

#### Relevance of the extracted sentences

At the level of the randomized trials, ExaCT correctly identified the reporting (reported or not reported) of a median 81.0% (*n* = 17/21) (IQR, 76.2% to 90.5% or 16/21 to 19/21) data elements. At the level of the individual data elements (Table 2), for 19.0% (*n* = 4/21) (eligibility criteria, enrollment end date, control arm(s), and primary outcome(s)) ExaCT correctly identified a solution (i.e., returned that a reported data element was “found”) for all randomized trials in which they were reported. For an additional 33.3% (*n* = 7/21) of data elements (first author name, date of publication, DOI, funding source, sample size, enrollment start date, and experimental arm[s]) solutions were identified for at least 90% of randomized trials in which they were reported. For an additional 23.8% (*n* = 5/21) of data elements (funding number, registration number, dose, duration of treatment, and secondary outcome[s]) solutions were identified for at least 75% of randomized trials in which they were reported. Solutions were less frequently correctly identified for the remaining 23.8% (*n* = 5/21) of data elements: early stopping (*n* = 2/4, 50.0%), route of administration (*n* = 14/29, 48.3%), frequency of administration (*n* = 28/43, 65.1%), primary outcome time point (*n* = 50/74, 67.6%), and secondary outcome time point (*n* = 23/54, 42.6%).

For data elements correctly identified as reported in the randomized trials, ExaCT provided five candidate sentences including a top sentence (“system suggestion”). At the level of the randomized trials, the top sentence was relevant for a median (IQR) 60.0% (50.0% to 75.0%) of data elements. At the level of the individual data elements, the top sentence reported for the registration number and early stopping were relevant in all solutions, and for the funding number in 90.9% (*n* = 20/22) of solutions. For an additional 33.3% (*n* = 6/18) of data elements (the first author name, date of publication, DOI, enrollment start date, route of administration, and frequency of administration) the top sentence was relevant among at least 80% of solutions. For an additional 22.2% (*n* = 4/18) of data elements (funding source, enrollment end date, primary outcome[s], and secondary outcome[s]) the top sentence was relevant among at least 70% of solutions. The top sentence was less frequently relevant among the solutions for the remaining 44.4% (*n* = 8/18) of data elements: control arm(s) (*n* = 49/75, 65.3%), secondary outcome time point (*n* = 15/23, 65.2%), duration of treatment (*n* = 25/41, 61.0%), dose (*n* = 19/32, 59.5%), experimental arm(s) (*n* = 43/74, 58.1%), primary outcome time point (*n* = 27/50, 54.0%), eligibility criteria (*n* = 38/75, 50.7%), and sample size (*n* = 32/68, 47.1%).

At the level of the randomized trials, at least one of the top five sentences was relevant for a median (IQR) 72.2% (63.6% to 84.2%) of data elements. At the level of the individual data elements, at least one of the top five sentences was relevant among all solutions for 23.8% (*n* = 5/21) (funding number, registration number, enrollment start date, early stopping, and route of administration). For an additional 16.7% (*n* = 3/18) of data elements (enrollment end date, frequency of administration, and secondary outcome[s]) at least one sentence was relevant across at least 90% of solutions. For an additional 27.8% (*n* = 5/18) of data elements (funding source, experimental arm[s], control arm[s], primary outcome[s], and secondary outcome time point) at least one sentence was relevant across at least 80% of solutions. For an additional 11.1% (*n* = 2/18) of data elements (duration of treatment and primary outcome time point) at least one sentence was relevant across at least 70% of solutions. At least one sentence was less frequently relevant among the solutions for the remaining 11.1% (*n* = 2/18) of data elements: eligibility criteria (*n* = 47/75, 62.7%) and sample size (*n* = 43/68, 63.2%).

### Relevance of the highlighted fragments

The relevance of the highlighted fragments within the relevant sentences is in Table 3. Seventy-nine percent (*n* = 124/157) of fragments for the funding source and 55.6% (*n* = 74/133) for the experimental arm(s) were considered relevant. For the remaining data elements, at least 81.5% of fragments were relevant.

**Table 3:** Relevance of the highlighted text fragments among relevant sentences^a^

Report section	Data element	Relevant sentences, n Total^b^	Fragments, n total^c^	Relevant fragments, n (%)^d^	Exact matches, n (%)^d^	Partial matches, n (%)^d^
**Meta information**	Funding source	79	157	*124 (79.0)*	*24 (15.3)*	100 (63.7)
	Funding number	35	54	*44 (81.5)*	27 (50.0)	17 (31.5)
	Registration number	63	104	104 (100.0)	103 (99.0)	*1 (1.0)*
**Enrollment**	Eligibility criteria	110	0	*0 (0.0)*	*0 (0.0)*	*0 (0.0)*
	Sample size	125	125	110 (88.0)	92 (73.6)	*18 (14.4)*
	Enrollment start date	55	51	51 (100.0)	43 (84.3)	8 (15.7)
	Enrollment end date	56	50	47 (94.0)	45 (90.0)	*2 (4.0)*
	Early stopping	7	3	3 (100.0)	1 (33.3)	2 (66.7)
**Intervention**	Experimental arm(s)	123	133	*74 (55.6)*	*15 (11.3)*	59 (44.4)
	Control arm(s)	121	62	55 (88.7)	34 (54.8)	21 (33.9)
	Route of administration	32	34	30 (88.2)	27 (79.4)	*3 (8.8)*
	Dose	50	77	70 (90.9)	26 (33.8)	44 (57.1)
	Frequency of administration	45	61	55 (90.1)	44 (72.1)	11 (18.0)
	Duration of treatment	57	56	*48 (85.7)*	35 (62.5)	13 (23.2)
**Outcome**	Primary outcome(s)	95	78	74 (94.9)	55 (70.5)	19 (24.4)
	Primary outcome time point	76	86	78 (90.7)	*28 (32.6)*	50 (58.1)
	Secondary outcome(s)	75	53	51 (96.2)	*16 (30.2)*	35 (66.0)
	Secondary outcome time point	43	53	51 (96.2)	20 (37.7)	31 (58.5)
**Summary measure**	Median (IQR), n	**57 (54)**	**59 (33)**	**53 (47 to 74)**	**27 (15 to 41)**	**18 (4 to 34)**
	Median (IQR), %	-	-	**90.4 (86.2 to 96.9)**	**52.4 (32.8 to 73.2)**	**28.0 (14.7 to 57.9)**

^a^ExaCT does not provide fragments for publication information. Data are shown for the remaining 18 data elements. Values in *italics* typeface fall at or below the limit of the lowest quartile

^b^Across all 75 trials, the number of relevant sentences among the 5 sentences reported within the solution for each data element

^c^Contained within sentences considered to be relevant by the human reviewers (column 3)

^d^Relevant fragments of those contained within sentences considered to be relevant by the human reviewers (denominator, column 4)

For 16.7% (*n* = 3/18) of data elements (registration number and enrollment start and end date), more than 80% of fragments were exact matches. For an additional 22.2% (*n* = 4/18) of data elements (sample size, route of administration, frequency of administration, primary outcome[s]) more than 70% were exact matches. Exact matches were less frequent among the remaining 61.1% (*n* = 11/18) of data elements: duration of treatment (*n* = 35/56, 62.5%), control arm(s) (*n* = 34/62, 54.8%), funding number (*n* = 27/54, 50.0%), secondary outcome time point (*n* = 20/53, 37.7%), dose (*n* = 26/77, 33.8%), early stopping (*n* = 1/3, 33.3%), primary outcome time point (*n* = 28/76, 32.6%), secondary outcome(s) (*n* = 16/53, 30.2%), funding source (*n* = 24/157, 15.3%), and experimental arm(s) (*n* = 15/133, 11.3%). Partial matches were most common among fragments provided in relevant sentences for the funding source (*n* = 100/157, 63.7%), early stopping (*n* = 2/3, 66.7%), dose (*n* = 44/77, 57.1%), primary outcome time point (*n* = 50/86, 58.1%), secondary outcome(s) (*n* = 35/53, 66.0%), and secondary outcome time point (*n* = 31/53, 58.5%).

### Overall relevance of the extracted solutions

At the level of the randomized trials, ExaCT provided a fully relevant solution for a median (IQR) 10 (9 to 12) (47.6% [42.9% to 57.1%]) data elements, a partially relevant solution for a median (IQR) 6 (5 to 8) (28.6% [23.8% to 38.1%]) data elements, and a fully irrelevant solution for a median (IQR) 4 (3 to 6) (19.0% [14.3% to 28.6%]) data elements. For the individual data elements (Table 4) a median (IQR) 36 (16 to 53) (48.0% [21.3% to 70.7%]) of all solutions (of a total 75 solutions for each data element across the randomized trials) were considered fully relevant, 22 (12 to 38) (29.3% [16.0% to 50.7%]) were considered partially relevant, and 13 (10 to 22) (17.3% [13.3% to 29.3%]) were considered fully irrelevant.

**Table 4:** Relevance of the extracted solutions

Report Section	Data Element	Fully Relevant Solutions, n (%)^a^	Partially Relevant Solutions, n (%)^a^	Fully Irrelevant Solutions, n (%)^a^
**Publication information**	First author name	63 (84.0)	*0 (0.0)*	12 (16.0)
	Date of publication	64 (85.3)	*0 (0.0)*	11 (14.7)
	Digital object identifier	62 (82.7)	*0 (0.0)*	13 (17.3)
**Meta information**	Funding source	*16 (21.3)*	38 (50.7)	21 (28.0)
	Funding number	52 (69.3)	13 (17.3)	*10 (13.3)*
	Registration number	62 (82.7)	*5 (6.7)*	*8 (10.7)*
**Enrollment**	Eligibility criteria	*0 (0.0)*	66 (88.0)	*9 (12.0)*
	Sample size	31 (41.3)	38 (50.7)	*6 (8.0)*
	Enrollment start date	34 (45.3)	15 (20.0)	26 (34.7)
	Enrollment end date	37 (49.3)	13 (17.3)	25 (33.3)
	Early stopping	70 (93.3)	*3 (4.0)*	*2 (2.7)*
**Intervention**	Experimental arm(s)	*10 (13.3)*	56 (74.7)	*9 (12.0)*
	Control arm(s)	*16 (21.3)*	49 (65.3)	*10 (13.3)*
	Route of administration	53 (70.7)	*12 (16.0)*	*10 (13.3)*
	Dose	36 (48.0)	22 (29.3)	17 (22.7)
	Frequency of administration	39 (52.0)	14 (18.7)	22 (29.3)
	Duration of treatment	22 (29.3)	25 (33.3)	28 (37.3)
**Outcome**	Primary outcome(s)	38 (50.7)	24 (32.0)	13 (17.3)
	Primary outcome time point	*7 (9.3)*	42 (56.0)	26 (34.7)
	Secondary outcome(s)	23 (30.7)	31 (41.3)	21 (28.0)
	Secondary outcome time point	*15 (20.0)*	27 (36.0)	33 (44.0)
**Summary measure**	Median (IQR), n	**36 (16 to 53)**	**22 (12 to 38)**	**13 (10 to 22)**
	Median (IQR), %	**48.0 (21.3 to 70.7)**	**29.3 (16.0 to 50.7)**	**17.3 (13.3 to 29.3)**

Values in *italics* typeface fall at or below the limit of the lowest quartile

^a^Out of a total 75 solutions per data element (i.e., one solution per data element, per trial). Partially correct solutions were those that included relevant information but either also included erroneous information or fell short of including all essential details

More than 80% of solutions were fully relevant for 29% (*n* = 6/21) of data elements: first author name, date of publication, DOI, registration number, and early stopping. The data elements for which the solutions were least frequently fully relevant included: control arm (*n* = 16/75, 21.3%), funding source (*n* = 16/75, 21.3%), secondary outcome time point (*n* = 15/75, 20.0%), experimental arm (*n* = 10/75, 13.3%), primary outcome time point (*n* = 7/75, 9.3%), and eligibility criteria (*n* = 0/75, 0.0%).

Accounting for both fully and partially relevant solutions, a median (IQR) 82.7% (70.7% to 86.7%) were at least partially relevant. More than 80% of solutions were at least partially relevant for 57.1% (*n* = 12/21) of data elements: first author name, date of publication, DOI, funding number, registration ID, eligibility criteria, sample size, early stopping, experimental arm(s), control arm(s), route of administration, and primary outcome(s). More than 70% of solutions were at least partially relevant for an additional 19.0% (*n* = 4/21) of data elements: funding source, dose, frequency of administration, and secondary outcome(s). For the remaining 23.8% (*n* = 5/21) of data elements, solutions that were at least partially relevant were less frequent: enrollment end date (*n* = 50/75, 66.7%), enrollment start date (*n *= 49/75, 65.3%), primary outcome time point (*n* = 49/75, 65.3%), duration of treatment (*n* = 47/75, 62.7%), secondary outcome time point (*n* = 42/75, 56.0%).

### C. Time Savings

It took the reviewers a median (IQR) 16.4 (14.3 to 19.8) minutes to manually extract the data from each randomized trial and an additional 8.0 (6.4 to 10.0) minutes for the second reviewer to complete the verification. The combined time to manually extract and verify the data from each randomized trial was a median (IQR) 24.7 (21.2 to 29.4) minutes. Overall, we spent 21.6 h manually extracting and 10.7 h verifying data from the 75 randomized trials, for a total workload of 32.3 h.

It took the reviewers a median (IQR) 13.8 (11.0 to 17.6) minutes to review and amend the automated extractions. This equates to a median 2.6 min faster compared with manual extraction by a single reviewer. Overall, we spent a total of 17.9 h extracting data from the 75 randomized trials with the assistance of ExaCT.

In the context of using the tool to assist the first reviewer in a pair (i.e., to expedite the first reviewer’s extractions), this equates to a median 3.7 h less time spent extracting data compared with manual extraction (17.9 h versus 21.6 h, 17.1% time savings across 75 randomized trials). The verification time (for the second reviewer, not measured) we assume, would remain constant. In the context of using the tool to replace the first reviewer in a pair (i.e., as a primary source for data extraction that would be validated by a human reviewer) this equates to a median 14.4 h less time spent extracting and verifying data compared with manual extraction and verification (17.9 h versus 32.3 h, 44.6% time savings across 75 randomized trials).

## Discussion

Across a sample of 75 randomized trials, ExaCT correctly identified the reporting (reported or not reported) of data elements more than 90% of the time for 52% of data elements (*n* = 11/21). For three (14%) data elements (route of administration, early stopping, secondary outcome time point), the tool correctly identified their reporting (reported or not reported) 50% of the time or less. Among the top five sentences presented for each solution, for 81% (*n* = 17/21) of data elements at least one sentence was relevant more than 80% of the time. For the remaining four data elements (eligibility criteria, sample size, duration of intervention, primary outcome time point) the relevance of the top five sentences was considerably less. For 83% (*n* = 15/18) of data elements, relevant fragments were highlighted among the relevant sentences more than 80% of the time. For the remaining three data elements (funding source, eligibility criteria, experimental arm) the highlighted fragments were more often irrelevant. Fully correct solutions were common (> 80%) for some data elements (first author name, data of publication, DOI, funding number, registration number, early stopping) but performance varied greatly (from 0% for eligibility criteria to 93% for early stopping). Solutions were most frequently (> 30%) fully irrelevant for enrollment start and end date, duration of treatment, and primary and secondary outcome time points. Using ExaCT to assist the first reviewer in a pair resulted in a modest time savings compared with manual extraction by one reviewer (17.9 h compared with 21.6 h, 17.1%). The time saved applies only to the small proportion of data elements that are typically extracted from randomized trials in the context of a systematic review, and only to the work of the first reviewer in a pair.

Our findings extend those published by Kiritchenko et al. in 2010 [13]. We are not aware of any other published evaluations of the ExaCT prototype (demo). For a sample of 50 drug trials, Kiritchenko et al. reported 80% precision (the proportion of returned instances that are truly relevant) and recall (the proportion of relevant instances returned by the system) for the system suggestion (top sentence); among 93% of solutions, at least one of the top five sentences was relevant [13]. Performance was substantially poorer only for the funding source, eligibility criteria, and primary outcome time point [13]. Precision and recall were more than 90% for extracted fragments. Across data elements, the solutions were fully correct 66% of the time [13]. We anticipated that performance in our evaluation would be poorer, given that the system was first evaluated only on drug trials [13] and our sample consisted of randomized trials unrestricted by intervention (only 24% were drug trials). We presumed, then, that the tool would have greater difficulty correctly identifying the experimental arm and details of the intervention (e.g., frequency of administration, route of administration). Indeed, we found that the top sentence was relevant across a median 78% of solutions, but results varied greatly across data elements (from 47% for the sample size to 100% for registration number and early stopping). Remarkably, performance was relatively similar for the top five sentences (relevant across a median 88% of solutions) and extracted fragments (relevant across a median 90% of relevant sentences). Solutions were considered fully correct with lesser frequency, likely because the top sentence was less often correct (48% vs. 66%). Performance could potentially be improved (to an unknown extent) via review-specific training of the tool (i.e., on a subset of included trials) by content experts; however, this is not an option in the demo.

Our findings suggest that using ExaCT to assist the first reviewer may be slightly more efficient than manual extraction by a single reviewer; however, before adopting semi-automated approaches to data extraction, gains in efficiency must be weighed against usability and the accuracy of the extractions. As we have demonstrated, substantially more time could be saved if the automated extractions could be used to fully replace the first reviewer; however, many review teams may not be comfortable adopting this approach. The majority of solutions required at least some editing (to sentence selection, the highlighted fragments, or both); thus, the automated extractions are likely not a suitable replacement for the first reviewer. Time was saved because the reviewers were often more quickly able to identify the location of relevant data in the full texts; however, the process otherwise often resembled manual extraction because the reviewers needed to add relevant data or make amendments based on what was found in the text. Reviewers must also account for the fact that the automated extractions were reflective only of information contained within the source document. Typically, reviewers would ensure the completeness of the extraction by using multiple sources, including the trial registry, associated publications, and supplementary files to complete the extraction [17]. As this is a common issue among automated data extraction systems, [12, 18] to support their utility more sophisticated systems that can incorporate data from multiple sources per randomized trial will be required.

### Strengths and limitations

To our knowledge this is the first study to externally and prospectively evaluate the performance of the ExaCT tool. We tested the tool on a heterogeneous sample of randomized trials of pediatric health interventions published during a one year period. As all of the randomized trials in the sample were published relatively recently (2017), the performance of the tool on older randomized trials (which presumably would be less well reported) may be worse. The findings may also not be generalizable to randomized trials in specific clinical areas. Reviewers who are less experienced with data extraction or the conduct of systematic reviews extract data slower than those with substantial experience in either area [19]. Since all of our reviewers were substantially experienced, our findings may not be generalizable to data extraction undertaken by less experienced review teams (it is possible that both the manual data extraction and verification, and the semi-automated extraction could take longer). Further research is required to determine how reviewer experience might affect time savings.

Although time was saved when ExaCT was used to assist with data extraction, the efficiency gained applies only to a small proportion of the data typically extracted from randomized trials for the purpose of a systematic review. The automatically extracted data elements are also arguably those more quickly and easily manually identified and extracted (e.g., compared with outcome data, for which identification and extraction is often more complex). It is always possible that a learning effect (i.e., increase in the pace of data extraction due to familiarity with the data extraction items) could have resulted in an overestimate of time savings; however, this is unlikely. Our reviewers were already highly experienced and completed pilot testing of the forms prior to formal data extraction. Since the process for reviewing and amending the automated extractions differed substantially from the process used to manually extract and verify data, any learnings from either process would not be directly transferable.

We did not formally evaluate the accuracy and completeness of the semi-automated data extractions compared with those manually extracted by the reviewers. As the accuracy and completeness of the extracted data have important implications with respect to the results and conclusions of systematic reviews, evaluations directly comparing manually and semi-automatically extracted data will help to inform how ExaCT and similar tools may most reliably be used. Specifically, it may be interesting to know whether the accuracy and completeness of the semi-automated extractions are more similar to a single reviewer’s manual extractions or to data manually extracted by one reviewer and verified by another. This would inform whether the tool may be better used to assist or fully replace the first reviewer in a pair.

## Conclusions

In this prospective evaluation, using ExaCT to assist the first reviewer in a pair to extract data from randomized trials was slightly more efficient compared with manual extraction. The tool was reliable for identifying the reporting (reported or not reported) of most data elements; however, the relevance of the system suggestion (top sentence) varied substantially across data elements. Among the top five sentences presented for each solution, for 81% of data elements at least one sentence was relevant more than 80% of the time. For 83% of data elements, relevant fragments were highlighted among the relevant sentences more than 80% of the time. Fully correct solutions were relatively infrequent for most data elements, with the exception of first author name, date of publication, DOI, funding number, registration number, early stopping. For other data elements, changes to sentence selection or the highlighted fragments were often required.

## Supplementary Information

**Additional file 1.**

**Additional file 2.**

**Additional file 3.**

**Additional file 4.**

## Data Availability

The datasets used and/or analyzed during the current study are available from the corresponding author on reasonable request at: https://doi.org/10.7939/DVN/9BQ1TK.

## Abbreviations

DOI: Digital object identifier
HTML: Hypertext markup language
ID: Identification number
IQR: Interquartile range
