# Supplementary material for: Creating efficiencies in the extraction of data from randomized trials: a prospective evaluation of a machine learning and text mining tool
Source: BMC Med Res Methodol. 2021 Aug 16;21:169. doi: 10.1186/s12874-021-01354-2 (PMC8369614; doi:10.1186/s12874-021-01354-2)
Supplement: Supplementary file 1 — Additional file 1. [file 12874_2021_1354_MOESM1_ESM.docx]

Additional File 1

File name: ExTRAKT – Additional File 1.docx

File format: Microsoft Word document (.docx)

Title of data: Search Strategy

Description of data: Search strategy used to identify the sample of trials

Additional File 1. Search Strategy

| Database | Date Searched | Number Retrieved |
| --- | --- | --- |
| Cochrane Central Register of Controlled Trials (Wiley) | February 19, 2020 | 17703 |
| Total |  | **17703** |

Strategy

#1 (Infant* or infancy or Newborn* or Baby* or Babies or Neonat* or Preterm* or Prematur* or Postmatur* or Child* or Schoolchild* or School age* or Preschool* or Kid or kids or Toddler* or Teen* or Boy* or Girl* or Minors* or Pubert* or Pubescen* or Prepubescen* or Pediatric* or Paediatric* or Peadiatric* or Nursery school* or Kindergar* or Primary school* or Secondary school* or Elementary school* or High school* or Highschool*):ti,ab,kw

#2 Adolesc*:ti,ab

#3 (Infant or Child or Minors or Puberty or Pediatrics or Schools):kw

#4 #1 or #2 or #3

#5 adolescent*:kw

#6 (adolescent* and (adult* or elderly or "middle aged" or "aged, 80 and over")):kw

#7 #6 not #4

#8 #4 or #5

#9 #8 not #7 with Publication Year from 2017 to 2017, in Trials
