# Supplementary material for: Creating efficiencies in the extraction of data from randomized trials: a prospective evaluation of a machine learning and text mining tool
Source: BMC Med Res Methodol. 2021 Aug 16;21:169. doi: 10.1186/s12874-021-01354-2 (PMC8369614; doi:10.1186/s12874-021-01354-2)

**Additional File 2**

**File name:** ExTRAKT – Additional File 2.docx

**File format:** Microsoft Word document (.docx)

**Title of data:** Summary of the Data Extraction and Analysis Protocol

**Description of data:** Visual representation of the data extraction and analysis protocol

Additional File 2. Summary of the Data Extraction and Analysis Protocol


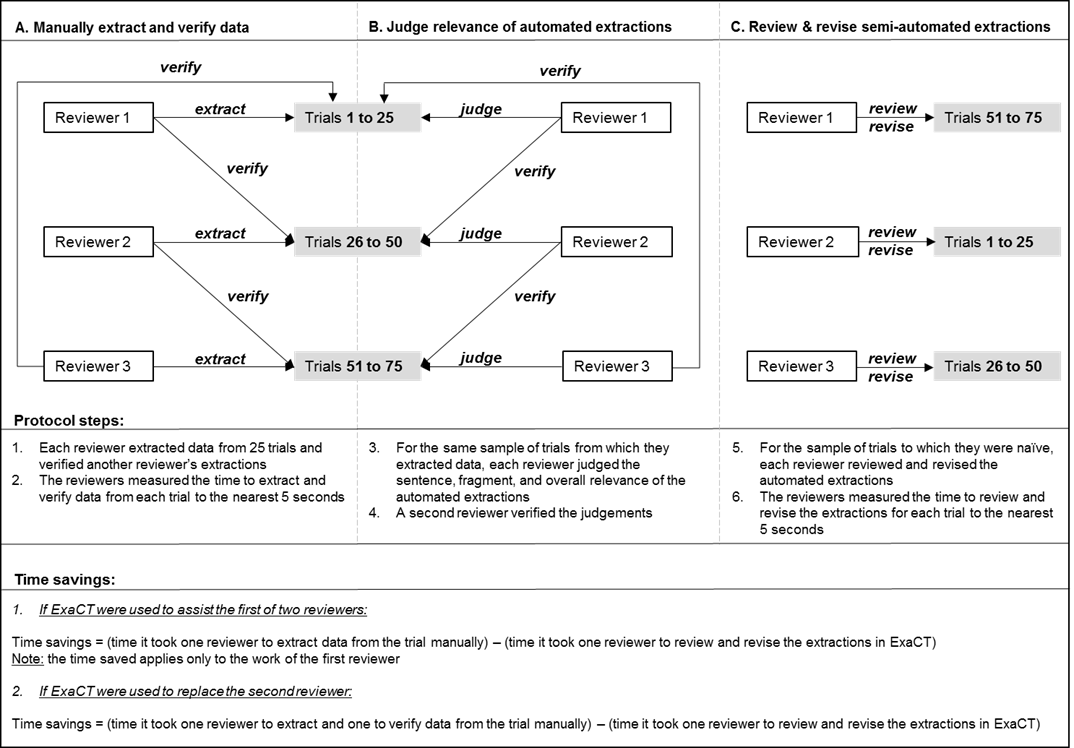

Supplement: Supplementary file 2 — Additional file 2. [file 12874_2021_1354_MOESM2_ESM.docx]
