# Supplementary material for: Creating efficiencies in the extraction of data from randomized trials: a prospective evaluation of a machine learning and text mining tool
Source: BMC Med Res Methodol. 2021 Aug 16;21:169. doi: 10.1186/s12874-021-01354-2 (PMC8369614; doi:10.1186/s12874-021-01354-2)
Supplement: Supplementary file 3 — Additional file 3. [file 12874_2021_1354_MOESM3_ESM.docx]

**Additional File 3**

**File name:** ExTRAKT – Additional File 3.docx

**File format:** Microsoft Word document (.docx)

**Title of data:** Examples of Relevant and Irrelevant Sentences, Fragments, and Solutions

**Description of data:** Examples of relevant and irrelevant sentences, fragments, and solutions

Additional File 3. Examples of Relevant and Irrelevant Sentences, Fragments, and Solutions

Quality of the Extracted Sentences

At the sentence level, for each data element the reviewers judged whether the top-ranked sentence was relevant (yes or no) and whether at least one sentence was relevant (even if it was not the top-ranked sentence; yes or no).

**Example of a relevant top-ranked sentence (Aboud 2017, data element:** experimental arm**)**

**Human reviewer’s verified extraction:** “Iodized salt as early as it was available”

**ExaCT’s extraction (fragments highlighted in yellow):**

| Sentence Ranking | Sentence |
| --- | --- |
| 1 | The study used a cluster randomized design in which clusters were defined as districts randomly assigned to receive iodized salt as early as it was available (intervention) or when market forces brought it in (control). |
| 2 | Sixty district clusters were randomized to receive iodized salt early at their markets with assistance from regular salt distributors or later as introduced by market forces. |
| 3 | The hypothesis was that iodized salt would enhance mental development outcomes for intervention children in rural villages randomized to receive it early compared with control children. |
| 4 | The intervention group received iodized salt provided by private companies around Lake Afdera, north east of Amhara. |
| 5 | Consequently the intervention group received approximately 4 to 6 months more exposure to iodized salt than controls; intervention children had approximately 8 to 10 months of iodized salt and control children had 4 to 6 months at endline. |

*Explanation:* the top-ranked sentence is relevant because it contains correct information about the experimental arm.

**Example of an irrelevant top-ranked sentence (Guven 2017, data element:** experimental arm**)**

**Human reviewer’s verified extraction**: “Various pulpotomy medicaments: (1) BD placed in pulp chamber and allowed to finish setting completely, followed by permanent restoration on the same session; (2) MTA-P + glass ionomer base place over the MTA; (3) PR-MTA placed in pulp chamber and condensed lightly with a moisted cotton pellet + glass ionomer base applied over the MTA; (4) 20% FS solution applied onto pulp stumps for 15s and after rinsing with water, ZOE base was placed.”

**ExaCT’s extraction:**

| Sentence Ranking | Sentence |
| --- | --- |
| 1 | Success Rates of Pulpotomies in Primary Molars Using Calcium Silicate-Based Materials: A Randomized Control Trial |
| 2 | This randomized clinical trial was conducted to examine and compare the effectiveness of pulpotomy in primary molars treated with calcium silicate-based materials: two MTA products (i.e., ProRoot MTA and MTA-P) and BD and FS as the control material. |
| 3 | At 12 months, PCA was observed in two teeth treated with BD and one tooth treated with MTA-P; those teeth showed PCO at 24 months as well. |
| 4 | K.C. Huth, E. Paschos, N. Hajek-Al-Khatar et al. Effectiveness of 4 pulpotomy techniques – Randomized controlled trial. Journal of Dental Research, vol. 84, no. 12, pp. 1144-1148, 2005. |
| 5 | The aim of this study was to evaluate and compare, both clinically and radiographically, the effects of calcium silicate-based materials (i.e., ProRoot MTA (PR-MTA), MTA-Plus (MTA-P), and Biodentine (BD)) and ferric sulfate (FS) in pulpotomy of primary molars. |

*Explanation:* the top-ranked sentence is irrelevant because it does not contain information about the nature of the experimental arms.

**Example of at least one relevant sentence (Papathomas 2017, data element:** experimental arm**)**

**Human reviewer’s verified extraction**: “Dialogic argumentation curriculum of four topics, including six electronic dialogs in peer pairs per topic, with an individual electronic dialog at the end of the school year. During topics 2, 3, and 4, at three of the six dialog sessions (1st, 3rd, and 5th), an adult substituted for an opposing peer pair.”

**ExaCT’s extraction (fragments highlighted in yellow):**

| Sentence Ranking | Sentence |
| --- | --- |
| 1 | Participants were randomly assigned to an experimental group or a comparison group (24 studies each, balanced across gender and drawn equally from the two classrooms). |
| 2 | During two class periods per week throughout the school year, all sixth graders at the school participated in a dialogic argumentation curriculum very similar to the one reported on by Kuhn and Crowell (2011) and Kuhn, Hemberger, and Khait (2016). |
| 3 | As such, the role of the other (or others in discourse involving more than two people) assumes a prominent place. |
| 4 | Constructivists may claim that argumentation serves cognitive development by providing engagement and practice that allows both participants to develop their reasoning skills via shared exercise. |
| 5 | In this case, in addition to the exercise provided by participation alone, will this interaction also offer the less capable participant benefit in terms of skill development? |

*Explanation:* the top-ranked sentence is irrelevant, but the second sentence contains correct information about the experimental arm.

Quality of the Extracted Fragments

At the fragment level, for each sentence that the reviewer considered relevant, they judged whether the highlighted text fragments were fully or at least partially relevant (yes or no). Fully relevant fragments were those that encompassed the full solution for the data element, without including additional irrelevant information or missing critical information. Partially relevant fragments were those that encompassed part of the solution, but either also included erroneous information or fell short of including all essential details.

**Example of a fully relevant fragment in a relevant sentence (Freedman 2017, data element:** experimental arm**)**

**Human reviewer’s verified extraction**: “In-clinic feeding of Impact(R) Peptide 1.5 (Nestle Health Science Inc) through digestive cartridge”

**ExaCT’s extraction (fragments highlighted in yellow):**

| Sentence Ranking | Sentence |
| --- | --- |
| 1 | After a 7-day washout period when participants received Peptamen 1.5 with their usual dose of PERT products but without digestive cartridge, participants crossed over and received EN through the opposite cartridge (placebo or digestive cartridge). |
| 2 | Participants were randomized to first receive EN through either digestive cartridge or placebo cartridge. |
| 3 | Increased Fat Absorption From Enteral Formula Through an In-line Digestive Cartridge in Patients With Cystic Fibrosis |
| 4 | Patients with CF receiving EN participated in a multicenter, randomized, double-blind, crossover trial with an open-label safety evaluation period. |
| 5 | Efficacy data were analyzed using differences in the plasma FA concentrations for 24 hours after a single EN feeding administered through either digestive cartridge or placebo cartridge. |

*Explanation:* the first two sentences are relevant because they contain correct information about the experimental arm. The fragment in the first sentence is fully relevant because it contains all necessary information about the data element, without including additional irrelevant information or missing critical information.

**Example of a partially relevant fragment in a relevant sentence (Freedman 2017, data element:** experimental arm**)**

**Human reviewer’s verified extraction:** “In-clinic feeding of Impact(R) Peptide 1.5 (Nestle Health Science Inc) through digestive cartridge”

**ExaCT’s extraction (fragments highlighted in yellow):**

| Sentence Ranking | Sentence |
| --- | --- |
| 1 | After a 7-day washout period when participants received Peptamen 1.5 with their usual dose of PERT products but without digestive cartridge, participants crossed over and received EN through the opposite cartridge (placebo or digestive cartridge). |
| 2 | Participants were randomized to first receive EN through either digestive cartridge or placebo cartridge. |
| 3 | Increased Fat Absorption From Enteral Formula Through an In-line Digestive Cartridge in Patients With Cystic Fibrosis |
| 4 | Patients with CF receiving EN participated in a multicenter, randomized, double-blind, crossover trial with an open-label safety evaluation period. |
| 5 | Efficacy data were analyzed using differences in the plasma FA concentrations for 24 hours after a single EN feeding administered through either digestive cartridge or placebo cartridge. |

*Explanation:* the first two sentences are relevant because they contain correct information about the experimental arm. The fragment in the second sentence is partially relevant because it contains relevant information about the experimental arm, but is missing important details (i.e., the name of the enteral nutrition product).

**Example of a fully irrelevant fragment in a relevant sentence (Razi 2017, data element:** experimental arm**)**

**Human reviewer’s verified extraction:** “Budesonide (1 mg/2 ml) with salbutamol nebules (0.15mg/kg/ dose, max. 5mg) driven by 100% oxygen at a flow of 6 L/min at 0, 20, and 40 min. Note: Both groups received one dose of intramuscularly methylprednisolone (1mg/kg/dose) at the onset of the treatment and salbutamol nebules at 80, 120, and 180 min”

**ExaCT’s extraction (fragments highlighted in yellow):**

| Sentence Ranking | Sentence |
| --- | --- |
| 1 | Children in the active treatment group were administered three doses of budesonide (1 mg/2 ml) with salbutamol nebules (0.15 mg/kg/dose, max. 5 mg) driven by 100% oxygen at a flow of 6 L/min at 0, 20, and 40 min and children in the control group received three doses of normal saline (2 ml) as a placebo as well as salbutamol. |
| 2 | In a study, which was conducted on 150 children who have moderate acute asthma exacerbation to examine the effects of different inhaled fluticasone doses, the authors did not demonstrate any improvement of SaO2 and PEF in the group who received three doses of salbutamol plus two doses of fluticasone 500 mcg/dose at 15 and 30 min after the first dose of salbutamol (accumulated dose of fluticasone = 1,000 mcg). |
| 3 | These beneficial effects have been reported only when patients received multiple ICS doses along with beta 2 agonists when compared with SCSs or placebo. |
| 4 | The object of this study was to determine whether high doses (total of 3 mg) of inhaled budesonide provide any additional benefits to a standardized treatment regimen that includes systemic steroids and salbutamol in preschool children who admitted to the ED with acute wheezing episodes. |
| 5 | The compared the effect of 1,500 g nebulized budesonide when added to standard acute asthma treatment (three doses of salbutamol, three doses of ipratropium bromide, and a single 2 mg/kg dose of prednisolone given at the beginning of therapy). |

*Explanation:* the first and fourth sentences are relevant because they contain correct information about the experimental arm. The fragment in the first sentence is fully irrelevant because it does not contain any relevant information about the experimental arm.

Overall Quality of the Extracted Solutions

‘Solutions’, which encompass both the extracted sentences and fragments, were considered fully relevant when the system identified a sentence with the target information as its top sentence and extracted the relevant fragments, or the system correctly reported the absence of the solution when it was not reported in the publication (i.e., returned a ‘not found’ solution). Solutions were partially relevant when the correct solution was present among the five sentences, but not (only) in the top sentence and/or the fragment selection in the sentence(s) was not entirely relevant. Solutions were irrelevant when none of the five suggested sentences contained relevant information pertaining to the data element.

**Example of a fully relevant solution (Indrio 2017, data element:** experimental arm**)**

**Human reviewer’s verified extraction:** “Freeze-dried L. reuteri DSM 17938 supplementation”

**ExaCT’s extraction (fragments highlighted in yellow):**

| Sentence Ranking | Sentence |
| --- | --- |
| 1 | Preterm newborns were randomly assigned to receive L. reuteri DSM 17938 supplementation or placebo by the use of a computer-generated randomization scheme. |
| 2 | Newborn were randomly allocated during the first 48 h of life to receive either daily probiotic (10.8 colony forming units (CFUs) of L. reuteri DSM 17938) or placebo for one month. |
| 3 | A total of 60 preterm newborns were randomly assigned to L. reuteri DSM 17938 or to the placebo group. |
| 4 | Efficacy and safety of available treatments for visceral leishmanlasis in Brazil: A multicenter, randomized, open label trial. |
| 5 | Women’s education level amplifies the effects of a livelihoods-based intervention on household wealth, child diet, and child growth in rural Nepal |

*Explanation:* the solution is fully correct because the top sentence contains correct information about the experimental arm, and the highlighted fragment contains all necessary information about the data element, without including additional irrelevant information or missing critical information.

**Example of a fully relevant solution (Papathomas 2017, data element:** funder**)**

**Human reviewer’s verified extraction:** “Not reported”

**ExaCT’s extraction:**

| Sentence ranking | Sentence |
| --- | --- |
| 0 | **Funder:** not found |
| 1 | The transfer task data are, of course, critical to the current study. |
| 2 | The productivity of socially shared cognition is observed in the current study. |
| 3 | Thus, the study supports the concept of apprenticeship as a mechanism of development of higher-order and specifically argumentative reasoning skills, a conclusion of both theoretical and applied significance. |
| 4 | In this case, in addition to the exercise provided by participation alone, will this interaction also offer the less capable participant benefit in terms of skill development? |
| 5 | Much of what apprentices acquire through their participation is intellectual in nature knowledge and understanding. |

*Explanation:* the solution is fully correct because the human reviewers did not identify the data element in the text, and ExaCT returned a ‘not found’ solution.

**Example of a partially relevant solution (Laskin 2017, data element:** experimental arm**)**

**Human reviewer’s verified extraction:** “More frequent, shorter hemodialysis (5 days per week for 2h25min, total=12 hours)”

**ExaCT’s extraction (fragments highlighted in yellow):**

| Sentence ranking | Sentence |
| --- | --- |
| 1 | Subjects were randomized to the treatment sequence: either 3 days per week conventional HD for four hours per treatment (12 hours total per week, control) followed by 5 days per week short, more frequent HD for two hours and 25 minutes per treatment (12 hours total per week, intervention), or vice versa. |
| 2 | Subjects were then randomized to the first treatment sequence (5 days versus 3 days per week HD, each for 12 hours total per week), during which three casual blood pressure measurements were obtained and averaged prior to each HD session, three days per week, regardless of which treatment frequency they were receiving. |
| 3 | In adults, the Frequent Hemodialysis Network (FHN) randomized trial demonstrated that an average of two extra in-center HD treatments was associated with improved survival and left ventricular hypertrophy. |
| 4 | Two subjects withdrew from the study early, both during 3 days per week treatment; one subject had completed 5 days per week HD but did not want to continue four hour treatments during the 3 days per week period and the second subject received a kidney transplant. |
| 5 | Short, frequent, 5 days per week in-center hemodialysis versus 3 days per week treatment: a randomized cross-over pilot trial through the Midwest Pediatric Nephrology Consortium |

*Explanation:* the solution is partially correct because the top sentence contains correct information about the experimental arm, but the highlighted fragment is relevant to the control arm, not the experimental arm.

**Example of a partially relevant solution (Gerceker 2017, data element:** experimental arm**)**

**Human reviewer’s verified extraction**: “Care bundle with 3M™ Tegaderm™ Chlorhexidine Gluconate (CHG) I.V. Securement Dressings”

**ExaCT’s extraction:**

| Sentence Ranking | Sentence |
| --- | --- |
| 1 | This study included 27 patients; 14 patients were randomly assigned to intervention and 13 patients to the control group. |
| 2 | To compare the effects of the care bundles including chlorhexidine dressing and advanced dressings on the catheter-related bloodstream infection (CRBSI) rates in pediatric hematology-oncology patients with central venous catheters (CVCs). |
| 3 | Prevention of central venous catheter-associated bloodstream infections in paediatric oncology patients using 70% ethanol locks: A randomised controlled multi-centre trial |
| 4 | Impact of flushing with aseptic non-touch technique using pre-filled flush or manually prepared syringes on central venous catheter occlusion and bloodstream infections in pediatric hemato-oncology patients: A randomized controlled study |
| 5 | The effects of chlorhexidine dressing on health care-associated infection in hospitalized patients: A meta-analysis |

*Explanation:* the solution is partially correct because the second sentence contains correct information about the experimental arm, but not the top sentence. Also, there were no highlighted fragments.

**Example of a fully irrelevant solution (Hashi 2017, data element:** experimental arm**)**

**Human reviewer’s verified extraction**: “Health education on key WASH messages and demonstration of hand washing with soap”

**ExaCT’s extraction:**

| Sentence Ranking | Sentence |
| --- | --- |
| 1 | Efficacy and Safety of Letibotulinum Toxin A for the Treatment of Dynamic Equinus Foot Deformity in Children with Cerebral Palsy: A Randomized Controlled Trial |
| 2 | J. 1964; 2 : 230233 [PMC free article] [PubMed] [Google Scholar] Lutry S.P., Agboatwalla M., Feikin D.R. Effect of handwashing on child health: a randomised controlled trial. |
| 3 | Lancet. 2005; 366 : 225233. [PubMed] [Google Scholar] Luby S.P., Agboatwalla M., Painter J. Combining drinking water treatment and hand washing for diarrhoea prevention, a cluster randomised controlled trial. |
| 4 | Efficacy and Safety of Letibotulinum Toxin A for the Treatment of Dynamic Equinu… |
| 5 | Lancet. 2005; 366 : 225233. [PubMed] [Google Scholar] [Ref list] |

*Explanation:* the solution is completely incorrect because none of the sentences contain any relevant information related to the experimental arm.
