# Supplementary material for: Creating efficiencies in the extraction of data from randomized trials: a prospective evaluation of a machine learning and text mining tool
Source: BMC Med Res Methodol. 2021 Aug 16;21:169. doi: 10.1186/s12874-021-01354-2 (PMC8369614; doi:10.1186/s12874-021-01354-2)
Supplement: Supplementary file 4 — Additional file 4. [file 12874_2021_1354_MOESM4_ESM.docx]

**Additional File 4**

**File name:** ExTRAKT – Additional File 4.docx

**File format:** Microsoft Word document (.docx)

**Title of data:** Sample of trials

**Description of data:** List of the included trials

Additional File 4. Sample of Trials

Aboud FE, Bougma K, Lemma T, Marquis GS. Evaluation of the effects of iodized salt on the mental development of preschool-aged children: a cluster randomized trial in northern Ethiopia. Matern Child Nutr. 2017;13(2):e12322. doi: 10.1111/mcn.12322.

Ammari WG, Obeidat N, Khater M, Sabouba A, Sanders M. Mastery of pMDI technique, asthma control and quality-of-life of children with asthma: a randomized controlled study comparing two inhaler technique training approaches. Pulm Pharmacol Ther. 2017;43:46‐54. doi: 10.1016/j.pupt.2017.02.002.

Anantasit N, Cheeptinnakorntaworn P, Khositseth A, Lertbunrian R, Chantra M. Ultrasound versus traditional palpation to guide radial artery cannulation in critically ill children: a randomized trial. J Ultrasound Med. 2017;36(12):2495‐501. doi: 10.1002/jum.14291.

Aras I, Pasaoglu A, Olmez S, Unal I, Tuncer AV, Aras A. Comparison of stepwise vs single-step advancement with the functional mandibular advancer in class II division 1 treatment. Angle Orthod. 2017;87(1):82‐7. doi: 10.2319/032416-241.1.

Bae DS, Valim C, Connell P, Brustowicz KA, Waters PM. Bivalved versus circumferential cast immobilization for displaced forearm fractures: a randomized clinical trial to assess efficacy and safety. J Pediatr Orthop. 2017;37(4):239‐46. doi: 10.1097/BPO.000000000000​0655.

Banupriya N, Bhat BV, Benet BD, Catherine C, Sridhar MG, Parija SC. Short term oral zinc supplementation among babies with neonatal sepsis for reducing mortality and improving outcome - a double-blind randomized controlled trial. Indian J Pediatr. 2018;85(1):5-9. doi: 10.1007/s12098-017-2444-8.

Ben-Pazi H, Cohen A, Kroyzer N, Lotem-Ophir R, Shvili Y, Winter G, et al. Clown-care reduces pain in children with cerebral palsy undergoing recurrent botulinum toxin injections- a quasi-randomized controlled crossover study. PloS One. 2017;12(4):e0175028. doi: 10.1371/journal.pone.0175028.

Blake MS, Waloszek JM, Raniti M, Simmons JG, Murray G, Blake L, et al. The SENSE Study: treatment mechanisms of a cognitive behavioral and mindfulness-based group sleep improvement intervention for at-risk adolescents. Sleep. 2017;40(6):1-11. doi: 10.1093/sleep​/zsx061.

Bonnet D, Berger F, Jokinen E, Kantor PF, Daubeney PEF. Ivabradine in children with dilated cardiomyopathy and symptomatic chronic heart failure. J Am Coll Cardiol. 2017;70(10):1262‐72. doi: 10.1016/j.jacc.2017.07.725.

Chang HJ, Hong BY, Lee SJ, Lee S, Park JH, Kwon JY. Efficacy and safety of letibotulinum toxin A for the treatment of dynamic equinus foot deformity in children with cerebral palsy: a randomized controlled trial. Toxins (Basel). 2017;9(8):252. doi: 10.3390/toxins9080252.

Darling A, McDonald CR, Urassa WS, Kain KC, Mwiru RS, Fawzi WW. Maternal dietary L-arginine and adverse birth outcomes in Dar es Salaam, Tanzania. Am J Epidemiol. 2017;186(5):603‐11. doi: 10.1093/aje/kwx080.

DiVasta AD, Feldman HA, Rubin CT, Gallagher JS, Stokes N, Kiel DP, et al. The ability of low-magnitude mechanical signals to normalize bone turnover in adolescents hospitalized for anorexia nervosa. Osteoporos Int. 2017;28(4):1255‐63. doi: 10.1007/s00198-016-3851-9.

Dray JB, Bowman, J, Campbell E, Freund M, Hodder R, Wolfenden L, et al. Effectiveness of a pragmatic school-based universal intervention targeting student resilience protective factors in reducing mental health problems in adolescents. J Adolesc. 2017;57:74‐89. doi: 10.1016​/j.adolescence.2017.03.009.

El-Chimi MS, Awad HA, El-Gammasy TM, El-Farghali OG, Sallam MT, Shinkar DM. Sustained versus intermittent lung inflation for resuscitation of preterm infants: a randomized controlled trial. J Matern Fetal Neonatal Med. 2017;30(11):1273‐8. doi: 10.1080/147670​58.2016.1210598.

Elkhayat HA, Aly RH, Elagouza IA, El-Kabarity RH, Galal YI. Role of P-glycoprotein inhibitors in children with drug-resistant epilepsy. Acta Neurol Scand. 2017;136(6):639‐44.

Fjørtoft T, Ustad T, Follestad T, Kaaresen PI, Øberg GK. Does a parent-administrated early motor intervention influence general movements and movement character at 3months of age in infants born preterm? Early Hum Dev. 2017;112:20‐4. doi: 10.1016/j.earlhumdev.20​17.06.008

Freedman S, Orenstein D, Black P, Brown P, McCoy K, Stevens J, et al. Increased fat absorption from enteral formula through an in-line digestive cartridge in patients with cystic fibrosis. J Pediatr Gastroenterol Nutr. 2017;65(1):97‐101. doi: 10.1097/MPG.00000000000​01617.

Freira S, Lemos MS, Williams G, Ribeiro M, Pena F, Machado MDC. Effect of motivational interviewing on depression scale scores of adolescents with obesity and overweight. Psychiatry Res. 2017;252:340‐5. doi: 10.1016/j.psychres.2017.03.020.

Fridenson-Hayo S, Berggren S, Lassalle A, Tal S, Pigat D, Meir-Goren N, et al. 'Emotiplay': a serious game for learning about emotions in children with autism: results of a cross-cultural evaluation. Eur Child Adolesc Psychiatry. 2017;26(8):979-992. doi: 10.1007/s00787-017-0968-0.

Gaesser AHK, O. C. A randomized controlled comparison of emotional freedom technique and cognitive-behavioral therapy to reduce adolescent anxiety: a pilot study. J Altern Complement Med. 2017;23(2):102‐8. doi: 10.1089/acm.2015.0316.

Gal S, Ramirez JI, Maguina P. Autologous fat grafting does not improve burn scar appearance: a prospective, randomized, double-blinded, placebo-controlled, pilot study. Burns. 2017;43(3):486‐9. doi: 10.1016/j.burns.2016.09.019.

Garnæs KK, Nyrnes SA, Salvesen K, Salvesen Ø, Mørkved S, Moholdt T. Effect of supervised exercise training during pregnancy on neonatal and maternal outcomes among overweight and obese women. Secondary analyses of the ETIP trial: a randomised controlled trial. PloS One. 2017;12(3):e0173937. doi: 10.1371/journal.pone.0173937.

Gerceker GO, Yardimci F, Aydinok Y. Randomized controlled trial of care bundles with chlorhexidine dressing and advanced dressings to prevent catheter-related bloodstream infections in pediatric hematology-oncology patients. Eur J Oncol Nurs. 2017;28:14‐20. doi: 10.1016/j.ejon.2017.02.008

Giaccone A, Zuppa AF, Sood B, Cohen MS, O'Byrne ML, Moorthy G, et al. Milrinone pharmacokinetics and pharmacodynamics in neonates with persistent pulmonary hypertension of the newborn. Am J Perinatol. 2017;34(8):749‐58. doi: 10.1055/s-0036-1597996.

Gottschlich MM, Mayes T, Khoury J, Kagan RJ. Clinical trial of vitamin D2 vs D3 supplementation in critically ill pediatric burn patients. JPEN J Parenter Enteral Nutr. 2017;41(3):412‐21. doi: 10.1177/0148607115587948.

Grooten I, Koot M, Van Der Post J, Ris-Stalpers C, Naaktgeboren C, Mol BW, et al. Early enteral tube feeding in optimizing treatment for hyperemesis gravidarum: the Maternal and Offspring outcomes after Treatment of HyperEmesis by Refeeding (MOTHER) randomised controlled trial. Am J Clin Nutr. 2017;106(3):812-820. doi: 10.3945/ajcn.117.158931.

Guven Y, Aksakal SD, Avcu N, Unsal G, Tuna EB, Aktoren O. Success rates of pulpotomies in primary molars using calcium silicate-based materials: a randomized control trial. Biomed Res Int. 2017;2017:4059703. doi: 10.1155/2017/4059703.

Hamelmann E, Bernstein JA, Vandewalker M, Moroni-Zentgraf P, Verri D, Unseld A, et al. A randomised controlled trial of tiotropium in adolescents with severe symptomatic asthma. Eur Respir J. 2017;49(1):1601100. doi: 10.1183/13993003.01100-2016.

Han D, Liu YG, Pan S, Luo Y, Li J, Ou-Yang C. Comparison of hemodynamic effects of sevoflurane and ketamine as basal anesthesia by a new and direct monitoring during induction in children with ventricular septal defect: a prospective, randomized research. Medicine (Baltimore). 2017;96(50):e9039. doi: 10.1097/MD.0000000000009039.

Handeland K, Oyen J, Skotheim S, Graff IE, Baste V, Kjellevold M, et al. Fatty fish intake and attention performance in 14-15 year old adolescents: FINS-TEENS - a randomized controlled trial. Nutr J. 2017;16(1):64. doi: 10.1186/s12937-017-0287-9.

Hashi A, Kumie A, Gasana J. Hand washing with soap and WASH educational intervention reduces under-five childhood diarrhoea incidence in Jigjiga District, Eastern Ethiopia: a community-based cluster randomized controlled trial. Prev Med Rep. 2017;6:361‐8. doi: 10.1016/j.pmedr.2017.04.011.

Indrio F, Riezzo G, Tafuri S, Ficarella M, Carlucci B, Bisceglia M, et al. Probiotic supplementation in preterm: feeding intolerance and hospital cost. Nutrients. 2017;9(9):965. doi: 10.3390/nu9090965.

Iserbyt P, Theys L, Ward P, Charlier N. The effect of a specialized content knowledge workshop on teaching and learning Basic Life Support in elementary school: a cluster randomized controlled trial. Resuscitation. 2017;112:17‐21. doi: 10.1016/j.resuscitation.201​6.11.023.

Karanja DMS, Awino EK, Wiegand RE, Okoth E, Abudho BO, Mwinzi PNM, et al. Cluster randomized trial comparing school-based mass drug administration schedules in areas of western Kenya with moderate initial prevalence of Schistosoma mansoni infections. PLoS Negl Trop Dis. 2017;11(10):e0006033. doi: 10.1371/journal.pntd.0006033

Kornmann MN, Christmann V, Gradussen CJW, Rodwell L, Gotthardt M, Van Goudoever JB, et al. Growth and bone mineralization of very preterm infants at term corrected age in relation to different nutritional intakes in the early postnatal period. Nutrients. 2017;9(12):1318. doi: 10.3390/nu9121318.

Lambrechts DA, de Kinderen RJ, Vles JS, de Louw AJ, Aldenkamp AP, Majoie HJ. A randomized controlled trial of the ketogenic diet in refractory childhood epilepsy. Acta Neurol Scand. 2017;135(2):231‐9. doi: 10.1111/ane.12592.

Laskin BL, Huang G, King E, Geary DF, Licht C, Metlay JP, et al. Short, frequent, 5-days-per-week, in-center hemodialysis versus 3-days-per week treatment: a randomized crossover pilot trial through the Midwest Pediatric Nephrology Consortium. Pediatr Nephrol. 2017;32(8):1423-1432. doi: 10.1007/s00467-017-3656-x.

Lisante TA, Nuñez C, Zhang P. Efficacy and safety of an over-the-counter 1% colloidal oatmeal cream in the management of mild to moderate atopic dermatitis in children: a double-blind, randomized, active-controlled study. J Dermatolog Treat. 2017;28(7):659‐67. doi: 10.1080/09546634.2017.1303569.

Locatelli F, Bernardo ME, Bertaina A, Rognoni C, Comoli P, Rovelli A, et al. Efficacy of two different doses of rabbit anti-T-lymphocyte globulin to prevent graft-versus-host disease in children with haematological malignancies transplanted from an unrelated donor: a multicentre, randomised, open-label, phase 3 trial. Lancet Oncol. 2017;18(8):1126‐36. doi: 10.1016/S1470-2045(17)30417-5.

Lotfi Y, Rezazadeh N, Moossavi A, Haghgoo HA, Rostami R, Bakhshi E, et al. Preliminary evidence of improved cognitive performance following vestibular rehabilitation in children with combined ADHD (cADHD) and concurrent vestibular impairment. Auris Nasus Larynx. 2017;44(6):700-707. doi: 10.1016/j.anl.2017.01.011.

Lundbye-Jensen J, Skriver K, Nielsen JB, Roig M. Acute exercise improves motor memory consolidation in preadolescent children. Front Hum Neurosci. 2017;11:182. doi: 10.3389/fnhum.2017.00182.

Manoj M, Satya Prakash MVS, Swaminathan S, Kamaladevi RK. Comparison of ease of administration of intranasal midazolam spray and oral midazolam syrup by parents as premedication to children undergoing elective surgery. J Anesth. 2017;31(3):351-357. doi: 10.1007/s00540-017-2330-6.

Martinon-Torres F, Safadi MAP, Martinez AC, Marquez PI, Torres JCT, Weckx LY, et al. Reduced schedules of 4CMenB vaccine in infants and catch-up series in children: immunogenicity and safety results from a randomised open-label phase 3b trial. Vaccine. 2017;35(28):3548-3557. doi: 10.1016/j.vaccine.2017.05.023.

Mayfield CA, Child S, Weaver RG, Zarrett N, Beets MW, Moore JB. Effectiveness of a playground intervention for antisocial, prosocial, and physical activity behaviors. J Sch Health. 2017;87(5):338‐45. doi: 10.1111/josh.12506.

McConnachie A, Haig C, Sinclair L, Bauld L, Tappin DM. Birth weight differences between those offered financial voucher incentives for verified smoking cessation and control participants enrolled in the Cessation in Pregnancy Incentives Trial (CPIT), employing an intuitive approach and a Complier Average Causal Effects (CACE) analysis. Trials. 2017;18(1):337. doi: 10.1186/s13063-017-2053-x.

Miklowitz DJ, Schneck CD, Walshaw PD, Garrett AS, Singh MK, Sugar CA, et al. Early intervention for youth at high risk for bipolar disorder: a multisite randomized trial of family-focused treatment. Early Interv Psychiatry. 2017;13(2):208-216. doi: 10.1111/eip.12463.

Miller LC, Joshi N, Lohani M, Rogers B, Mahato S, Ghosh S, et al. Women's education level amplifies the effects of a livelihoods-based intervention on household wealth, child diet, and child growth in rural Nepal. Int J Equity Health. 2017;16(1):183. doi: 10.1186/s12939-017-0681-0.

Moody KM, Baker RA, Santizo RO, Olmez I, Spies JM, Buthmann A, et al. A randomized trial of the effectiveness of the neutropenic diet versus food safety guidelines on infection rate in pediatric oncology patients. Pediatr Blood Cancer. 2018;65(1). doi: 10.1002/pbc.26711.

Muratori P, Bertacchi I, Giuli C, Nocentini A, Lochman JE. Implementing coping power adapted as a universal prevention program in Italian primary schools: a randomized control trial. Prev Sci. 2017;18(7):754‐61. doi: 10.1007/s11121-016-0715-7.

O'Sullivan A, Fitzpatrick N, Doyle O. Effects of early intervention on dietary intake and its mediating role on cognitive functioning: a randomised controlled trial. Public Health Nutr. 2017;20(1):154‐64. doi: 10.1017/S1368980016001877.

Papathomas L, Kuhn D. Learning to argue via apprenticeship. J Exp Child Psychol. 2017;159:129‐39. doi: 10.1016/j.jecp.2017.01.013.

Pastor-Villaescusa B, Canete MD, Caballero-Villarraso J, Hoyos R, Latorre M, Vazquez-Cobela R, et al. Metformin for obesity in prepubertal and pubertal children: a randomized controlled trial. Pediatrics. 2017;140(1): e20164285. doi: 10.1542/peds.2016-4285.

Paz Castro R, Haug S, Kowatsch T, Filler A, Schaub MP. Moderators of outcome in a technology-based intervention to prevent and reduce problem drinking among adolescents. Addict Behav. 2017;72:64‐71. doi: 10.1016/j.addbeh.2017.03.013.

Porter S, McConnell T, McLaughlin K, Lynn F, Cardwell C, Braiden HJ, et al. Music therapy for children and adolescents with behavioural and emotional problems: a randomised controlled trial. J Child Psychol Psychiatry. 2017;58(5):586‐94. doi: 10.1111/jcpp.12656.

Rajavi Z, Feizi M, Naderi A, Sabbaghi H, Behradfar N, Yaseri M, et al. Graded versus ungraded inferior oblique anterior transposition in patients with asymmetric dissociated vertical deviation. J AAPOS. 2017;21(6):476‐9.e1. doi: 10.1016/j.jaapos.2017.07.213.

Razi CH, Cörüt N, Andiran N. Budesonide reduces hospital admission rates in preschool children with acute wheezing. Pediatr Pulmonol. 2017;52(6):720‐8. doi: 10.1002/ppul.23667.

Romanzini LP, Dos Santos AA Nunes ML. Characteristics of sleep in socially vulnerable adolescents. Eur J Paediatr Neurol. 2017;21(4):627-634. doi: 10.1016/j.ejpn.2016.12.013.

Romero GAS, Costa DL, Costa DL, Costa CHN, de Almeida RP, de Melo EV, et al. Efficacy and safety of available treatments for visceral leishmaniasis in Brazil: a multicenter, randomized, open label trial. PLoS Negl Trop Dis. 2017;11(6):e0005706.

Rowe SM, Daines C, Ringshausen FC, Kerem E, Wilson J, Tullis E, et al. Tezacaftor-ivacaftor in residual-function heterozygotes with cystic fibrosis. N Engl J Med. 2017;377(21):2024‐35. doi: 10.1056/NEJMoa1709847.

Senders SD, Bundick ND, Li J, Zecca C, Helmond FA. Evaluation of immunogenicity and safety of VARIVAX^TM^ New Seed Process (NSP) in children. Hum Vaccin Immunother. 2018:14(2):442-449. doi: 10.1080/21645515.2017.1388479.

Sharpe HP, Patalay P, Vostanis P, Belsky J, Humphrey N, Wolpert M. Use, acceptability and impact of booklets designed to support mental health self-management and help seeking in schools: results of a large randomised controlled trial in England. Eur Child Adolesc Psychiatry. 2017;26(3):315‐24. doi: 10.007/s00787-016-0889-3.

Spektor Z, Pumarola F, Ismail K, Lanier B, Hussain I, Ansley J, et al. Efficacy and safety of ciprofloxacin plus fluocinolone in otitis media with tympanostomy tubes in pediatric patients a randomized clinical trial. JAMA Otolaryngol Head Neck Surg. 2017;143(4):341‐9. doi: 10.1001/jamaoto.2016.3537.

Thurman TR, Nice J, Taylor TM, Luckett B. Mitigating depression among orphaned and vulnerable adolescents: a randomized controlled trial of interpersonal psychotherapy for groups in South Africa. Child Adolesc Ment Health. 2017;22(4):224‐31. doi:10.1111/camh.12241.

Tonguet-Papucci A, Houngbe F, Huybregts L, Ait-Aissa M, Altare C, Kolsteren P, Huneau J. Unconditional seasonal cash transfer increases intake of high-nutritional-value foods in young burkinabe children: results of 24-hour dietary recall surveys within the Moderate Acute Malnutrition Out (MAM'Out) randomized controlled trial. J Nutr. 2017;147(7):1418‐25. doi: 10.3945/jn.116.244517.

Urbancikova I, Prymula R, Goldblatt D, Roalfe L, Prymulova K, Kosina P. Immunogenicity and safety of a booster dose of the 13-valent pneumococcal conjugate vaccine in children primed with the 10-valent or 13-valent pneumococcal conjugate vaccine in the Czech Republic and Slovakia. Vaccine. 2017;35(38):5186-5193. doi: 10.1016/j.vaccine.2017.07.103.

Wei C, Allen RJ, Tallis PM, Ryan FJ, Hunt LP, Shield JP, et al. Cognitive behavioural therapy stabilises glycaemic control in adolescents with type 1 diabetes-Outcomes from a randomised control trial. Pediatr Diabetes. 2018;19(1):106-113. doi: 10.1111/pedi.12519.

Wei X, Zhang Z, Walley JD, Hicks JP, Zeng J, Deng S, et al. Effect of a training and educational intervention for physicians and caregivers on antibiotic prescribing for upper respiratory tract infections in children at primary care facilities in rural China: a cluster-randomised controlled trial. Lancet Glob Health. 2017;5(12):e1258‐e67. doi: 10.1016/S2214-109X(17)30383-2.

Whittaker R, Stasiak K, McDowell H, Doherty I, Shepherd M, Chua S, et al. MEMO: an mHealth intervention to prevent the onset of depression in adolescents: a double-blind, randomised, placebo-controlled trial. J Child Psychol Psychiatry. 2017;58(9):1014‐22. doi: 10.1111/jcpp.12753.

Winkler P, Janoušková M, Kozeny J, Pasz J, Mlada K,Weissova A. Short video interventions to reduce mental health stigma: a multi-centre randomised controlled trial in nursing high schools. Soc Psychiatry Psychiatr Epidemiol. 2017;52(12):1549‐57. doi: 10.1007/s00127-017-1449-y

Wong JMW, Ebbeling CB, Robinson L, Feldman HA, Ludwig DS. Effects of advice to drink 8 cups of water per day in adolescents with overweight or obesity a randomized clinical trial. JAMA pediatr. 2017;171(5):e170012. doi: 10.1001/jamapediatrics.2017.0012.

Wu YJ, Wu WF, Hung CW, Ku MS, Liao PF, Sun HL, et al. Evaluation of efficacy and safety of Lactobacillus rhamnosus in children aged 4-48 months with atopic dermatitis: an 8-week, double-blind, randomized, placebo-controlled study. J Microbiol Immunol Infect. 2017;50(5):684‐92. doi: 10.1016/j.jmii.2015.10.003.

Yuen VM, Li BL, Cheuk DK, Leung MKM, Hui TWC, Wong IC, et al. A randomised controlled trial of oral chloral hydrate vs. intranasal dexmedetomidine before computerised tomography in children. Anaesthesia. 2017;72(10):1191‐5. doi: 10.1111/anae.13981.

Zambrano LD, Priest JW, Ivan E, Rusine J, Nagel C, Kirby M, et al. Use of serologic responses against enteropathogens to assess the impact of a point-of-use water filter: a randomized controlled trial in western province, Rwanda. Am J Trop Med Hyg. 2017;97(3):876‐87. doi: 10.4269/ajtmh.16-1006.

Zhou Z, Chen T, Jin L, Zheng D, Chen S, He M, et al. Self-refraction, ready-made glasses and quality of life among rural myopic Chinese children: a non-inferiority randomized trial. Acta Ophthalmol. 2017;95(6):567‐75. doi: 10.1111/aos.13149.

Zhu Y, Lin J, Long H, Ye N, Huang R, Yang X, et al. Comparison of survival time and comfort between 2 clear overlay retainers with different thicknesses: a pilot randomized controlled trial. Am J Orthod Dentofacial Orthop. 2017;151(3):433‐9. doi: 10.1016/j.ajodo.2016.10.019.
